# Anticipated Self and Public Stigma in Suicide Prevention Professionals

**DOI:** 10.3389/fpsyt.2022.931245

**Published:** 2022-06-28

**Authors:** Saška Roškar, Domen Kralj, Karl Andriessen, Karolina Krysinska, Matej Vinko, Anja Podlesek

**Affiliations:** ^1^Department for Health Research and Development, National Institute of Public Health, Ljubljana, Slovenia; ^2^Centre for Mental Health, Melbourne School of Population and Global Health, The University of Melbourne, Melbourne, VIC, Australia; ^3^Department of Psychology, Faculty of Arts, University of Ljubljana, Ljubljana, Slovenia

**Keywords:** anticipated stigma, self-stigma, mental illness, help-seeking, suicidal behavior, suicidologists

## Abstract

**Background:**

Stigma about mental illness—both public and self—is one of the most important factors hindering help-seeking. Stigma can occur during an acute episode of mental illness or be anticipatory. One group affected by stigma, but often neglected, is mental health professionals. This study examined the anticipated form of mental-illness and help-seeking self-stigma and the anticipated form of public stigma of suicidal behavior among members of the International Association for Suicide Prevention. We hypothesized that suicidologists with a history of suicidality or mental illness would anticipate greater stigma from the public and self.

**Methods:**

The study received ethical approval from the Commission for Medical Ethics of the Republic of Slovenia. Data from 83 participants who completed an online survey (February to May 2020) with informed consent were analyzed using path analysis. We tested a model predicting help-seeking self-stigma based on (i) personal experience of mental illness using anticipated self-stigma of mental illness as a mediating variable and (ii) history of suicidal behavior using anticipated public stigma of suicidal behavior as a mediating variable.

**Results:**

Personal experience of mental illness predicted anticipation of self-stigma of mental illness (β = 0.26). History of suicidality predicted anticipation of public stigma of suicidal behavior (β = 0.29). Anticipated self-stigma of mental illness proved to be a stronger predictor of help-seeking self-stigma (β = 0.40) than anticipated public stigma of suicidal behavior (β = 0.07).

**Conclusions:**

It is important to intentionally support the mental health of suicide prevention professionals, as they are not immune to mental illness or various types of stigma. Because our sample was small and diverse, further research to better understand stigma concepts in this population is warranted.

## Introduction

Mental illness accounts for 7% of the global disease burden and 19% of years lived with disability (1). It, directly and indirectly, affects various forms of premature mortality, including suicide. Although effective treatments are available for various mental illnesses and suicidality, the treatment and help-seeking gaps remain high (2). The treatment gap is primarily due to structural barriers, such as access to help, unrecognized mental illness, or unrecognized suicidal behavior, whereas attitudinal barriers and associated stigma contribute substantially to the help-seeking gap (3, 4).

Stigma is a multidimensional phenomenon characterized by labeling, stereotyping, and separation, leading to loss of status and discrimination (5, 6). Stigma, especially when it occurs in the context of mental illness, can have even more harmful effects than the mental illness itself (7) and can be a risk factor for suicide (8).

Stigma can be broadly divided into two types: (i) public stigma, i.e., the way a person perceives public attitudes and opinions about mental illness and people with such illnesses, and (ii) self-stigma, i.e., internalized public stigma, which is composed of the attitudes and opinions that affected individuals have about themselves and their reference group (9, 10). Self-stigma is related to low self-esteem and low self-worth and can be divided into mental illness stigma and help-seeking stigma (10, 11), related yet independent constructs (10). Similarly, suicide-related stigma can be divided into public stigma and self-stigma (12). The public stigma of suicide involves labeling suicidal individuals as weak, irresponsible, selfish, and unable to cope with their problems. In contrast, the self-stigma of suicide refers to the concealment of suicidal behavior and feelings of shame among those who have attempted suicide (13).

The relationship between age, gender, and various concepts of stigma remains unclear, as the literature does not provide consistent results. Some studies found that older adults report less public stigma and self-stigma related to mental illness (14). In contrast, a systematic review and meta-analysis (15) found that age was not significantly associated with the self-stigma of mental illness. Regarding the influence of gender, some studies found that men tended to have higher public stigma and self-stigma associated with psychological help-seeking (16), whereas other found that help-seeking self-stigma was significantly higher in women than in men (17).

Stigma can be experienced and internalized during an acute episode of mental illness, but it can also be anticipated. Anticipated stigma refers to the belief and expectation that one will face prejudice, discrimination, stereotyping, and devaluation from others in the future if others know about their issue (18, 19). Anticipated stigma is a strong predictor of psychological distress and predicts people avoiding or underutilizing needed health care services (19). Self-stigmatizing beliefs, either anticipated or directly experienced, are associated with withdrawal from social support, rejection of help, and avoidance of treatment (20).

Stigma affects not only the general population but also mental health professionals who are vulnerable to burnout (21, 22), mental illness, such as depression (23, 24), and suicidal behavior (25). Professionals' mental health knowledge does not make them immune to such conditions (26, 27). On the contrary, their prior experiences of adversity, distress, and mental health problems, may be one of the reasons they pursue a career in mental health (28). According to studies, mental health professionals tend not to seek out the services they provide (27). They are more likely to disclose in their social circles than in work circles (28). The reason for this may be that they are trained to help others in distress and therefore tend to have high expectations of themselves (26, 29). This combination may reinforce the process of denial about one's psychological distress, shame, and hesitation to seek help (26, 29). They may fear being perceived as less competent by the public and colleagues, or even lose their license (27), and therefore avoid disclosing problems, asking for help, or approaching a colleague they believe is struggling (30, 31). People with mental illness face stigma and may avoid disclosing their mental health issues, which can be even more apparent when working in the mental health field. In addition, many stigmatizing attitudes toward people with mental illness come from mental health professionals, which can contribute to not admitting personal mental health struggles and seeking professional help (32). Delaying seeking help can have a detrimental effect on mental health and increase the risk for suicidal behavior. For example, studies have found that 61–82% of psychologists had a lifetime prevalence of depressive symptoms (23, 24, 33) and 42% experienced anxiety (33). Pope and Tabachnick (23) found that 29% of therapists had suicidal ideation, and 4% had attempted suicide. Studies have also shown a higher risk of suicide among medical professionals (34–36). The ratio of suicide rates among physicians compared to the general population is 1.4 for male physicians and 2.27 for female physicians (37, 38).

Considering that suicide prevention professionals are a specific type of mental health professionals and as any other population may be at risk for developing mental illness and suicidal behavior—particularly because distress of working with suicidal clients may pose an additional risk to their mental health (27)—it is important to understand the factors that hinder or facilitate help-seeking in this population. To the best of our knowledge, anticipated self and public stigma in suicidologists has not yet been studied. Our study represents the first attempt to illuminate this important issue. Its purpose was to examine various types of stigma and their predictors among suicide prevention professionals. We examined the relationships between their age, gender, past or present mental health problems, personal suicide experience, years being active in suicidology, anticipated self-stigma of mental health and help-seeking, and anticipated public stigma of suicidal behavior.

## Materials and Methods

### Participants and Procedure

Suicide prevention professionals come from a variety of professional and academic backgrounds. The International Association for Suicide Prevention (IASP) brings together academics, mental health professionals, and crisis workers dedicated to preventing suicidal behavior and mitigating its effects. Members of IASP are diverse according to their work type (e.g., clinicians encounter the topic of suicidality differently from researchers and academics). However, we believe that professionals deciding to join IASP have a specific relation to the topic of suicidality compared to non-members. They likely have more knowledge about it and show more interest, making the group relatively homogeneous despite having different backgrounds. We considered IASP members to be experts in suicide prevention and thus an appropriate group for this study.

The study received ethical clearance from the Commission for Medical Ethics of the Republic of Slovenia (Ref. No. 0120-609/2019/5), and it conformed to the Declaration of Helsinki provisions in 1995.

We invited potential participants via the IASP mailing list to participate in an online survey (in English language). An invitation was sent to 518 international e-mail addresses in the IASP database (all addresses available at the survey time). The survey was constructed on platform 1KA (39). The introduction page explained the survey's objectives, and by proceeding with the survey, participants confirmed that they were giving their informed consent to participate anonymously and have their data used in the research. The survey was active from 24 February to 24 May 2020. We mailed two reminder e-mails to the potential participants.

Three hundred three individuals accessed the survey. Out of them, 190 chose not to participate, 24 partially completed the survey, and 89 participants completed the survey (17% response rate). We excluded six participants with education atypical for suicide prevention professionals from the analysis. In the final sample (*N* = 83; 51 females, 32 males), most participants were mental health professionals (psychiatrists 27%, clinical psychologists 20%, and psychologists 10%), followed by teachers/professors (8%), sociologists (6%), health professionals (medical doctors 3%, nurses 2%), and allied (health) professionals (social workers, public health researchers, epidemiologists, etc., 23% in total).

### Instruments

Participants reported on sociodemographic data (age, gender, profession, years of activity in the field of suicidology) and personal experience with mental illness (ever having experienced or currently experiencing mental health problems) and suicidality (suicide ideation and attempt). To provide at least a minimum of intervention to those who were in distress at the time of the survey, we included the following disclaimer: “If you experience distress, we recommend that you speak to someone close (e.g., a family member, a friend, a colleague) or, if necessary, contact local telephone helplines, a doctor, or a mental health professional.” The disclaimer was presented in the Introduction section and again at the end of the survey.

To capture both subtypes of self-stigma related to mental illness, we used two questionnaires: The Self-Stigma of Mental Illness—SSOMI (11) and The Self-Stigma of Seeking Help—SSOSH (40). The authors refer to their questionnaires as instruments for measuring (experienced) self-stigma. However, because the items are phrased in terms of “would” (e.g., I would feel inadequate if I had a mental illness; I would feel inadequate if I went to a therapist for psychological help), we considered the instruments to measure anticipated self-stigma. Both questionnaires consist of 10 items rated on a 5-point Likert scale (1–strongly disagree; 5–strongly agree). The final score of both questionnaires ranges from 10 to 50, with higher scores indicating higher levels of self-stigma. In our study, both questionnaires showed good internal consistency. The Cronbach's alpha coefficient was 0.90 for SSOMI and 0.85 for SSOSH.

To assess the stigma related to suicide, we used the Personal Suicide Stigma Questionnaire–PSSQ (41). The questionnaire consists of 16 items rated on a 5-point scale (1–never; 5–very often). The final score ranges from 16 to 80 points, with a higher score indicating higher stigma. The original version of the questionnaire was designed to measure the responses that individuals with personal experience of suicidal behavior receive and perceive from their social environment. For our study, we modified the questions to apply to individuals with and without personal experience of suicide. The modified questions were asked in the “as if” form. For example, the original statement reads: “I have been treated as less competent by others when they learned about my suicidal thoughts or behavior.” In contrast, the modified version reads: “I would be treated as less competent by others if they learned about my suicidal thoughts or behavior.” Therefore, we considered the modified version to measure the anticipated public stigma of suicidal behavior. The modified version of the questionnaire showed good internal consistency (Cronbach's α = 0.93).

## Results

Table 1 presents the descriptive statistics of the sociodemographic variables, personal mental health history variables, and scores on the stigma scales. Approximately half of the sample reported mental health problems (past or present), and one-third reported suicidality (either suicidal ideation or attempt) in the past. The most prevalent reported mental health problem was depression (33%), followed by anxiety (24%). Of 42 (51%) participants who had experienced mental health issues, 9 (21%) reported not seeking professional help. Of 31 (37%) who had experienced suicidal ideation, 13 (42%) reported not seeking help for that.

**Table 1:** Descriptive statistics for the examined variables (*N* = 83).

**Variable**	***M* (*p*)**	**SD**	**Skewness**	**Kurtosis**
1. Gender	0.61^a^	–	–	–
2. Age	51.77	13.36	0.15	−0.74
3. Years active	17.15	11.94	0.83	0.05
4. Mental health issues	0.51^b^	–	–	–
5. Suicidal behavior experience	0.37^c^	–	–	–
6. PSSQ	46.75	12.25	−0.11	−0.38
7. SSOMI	30.38	8.04	−0.36	−0.20
8. SSOSH	20.19	6.88	0.27	−0.71

*PSSQ, the Personal Suicide Stigma Questionnaire (anticipation form) scale score; SSOMI, the Self-Stigma of Mental Illness scale score; SSOSH, the Self-Stigma of Seeking Help scale score*.

*All SD values reported in this paper were calculated as estimates of population σ*.

*^a^Proportion of women. ^b^Proportion of participants with mental health issues in the past or at present. ^c^Proportion of participants with personal suicidal behavior experience*.

Table 2 shows the correlations between the variables. Low correlations of gender with the personal stigma of suicidal behavior and self-stigma of mental illness were observed, with women showing slightly higher stigma. Age and years active in suicidology did not correlate with different types of anticipated stigma. Personal history of mental health problems was associated with anticipated self-stigma of mental illness. Participants with such history (*n* = 42, *M* = 32.83, *SD* = 7.79) showed higher anticipated self-stigma of mental illness than those without such history (*n* = 41, *M* = 28.78, *SD* = 7.86). Personal history of mental health problems was also associated with suicidal behavior and anticipated suicide-behavior public stigma (Table 2). There was a small positive correlation between the presence of suicidal experience and anticipated public stigma of suicidal behavior. The latter was higher in participants with suicidal experience (*n* = 31, *M* = 50.13, *SD* = 12.79) and lower in participants with no such experience (*n* = 52, *M* = 44.73, *SD* = 11.57).

**Table 2:** Correlations between the examined variables (*N* = 83).

**Variable**	**1**	**2**	**3**	**4**	**5**	**6**	**7**
1. Gender							
2. Age	−0.32**						
3. Years active	−0.34**	0.80***					
4. Mental health issues	0.26*	−0.23*	−0.23*				
5. Suicidal behavior experience	0.15	−0.15	−0.10	0.41***			
6. PSSQ	0.26*	0.00	−0.16	0.29**	0.21		
7. SSOMI	0.26*	−0.15	−0.14	0.25*	0.23*	0.45***	
8. SSOSH	0.10	0.07	0.03	0.01	0.07	0.25*	0.46***

*PSSQ, the Personal Suicide Stigma Questionnaire (anticipation form) scale score; SSOMI, the Self-Stigma of Mental Illness scale score; SSOSH, the Self-Stigma of Seeking Help scale score*.

*Pearson correlation coefficients were used for pairs of interval variables and phi-coefficients for pairs of binary variables*.

^*^p < 0.05. ^**^p < 0.01. ^***^p < 0.001.

**Figure 1:**
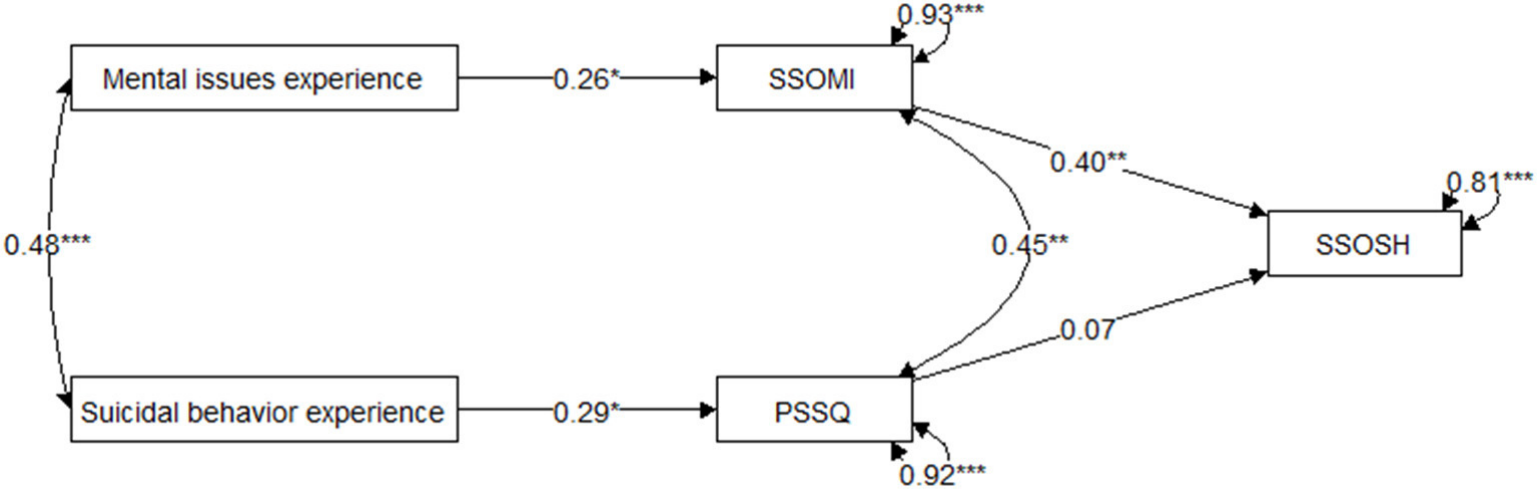
Model Predicting Anticipated Help-Seeking Self-Stigma Based on Personal History Variables With Anticipated Self-Stigma for Mental Illness and Anticipated Public Stigma for Suicidal Behavior as Mediators. PSSQ, the Personal Suicide Stigma Questionnaire (anticipation form) scale score; SSOMI, the Self-Stigma of Mental Illness scale score; SSOSH, the Self-Stigma of Seeking Help scale score. ^*^p < 0.05. ^**^p < 0.01. ^***^p < 0.001.

We further examined the relationships between the anticipated mental-health and help-seeking self-stigma, the anticipated public stigma of suicidal behavior, and personal experiences of mental health issues and suicidal behavior. Due to the low number of data available, we tested a simple model in which a history of mental health issues and suicidal behavior predicted the anticipation of self-stigma for mental health and public stigma for suicidal behavior, respectively. Both types of stigma were used as predictors of anticipated self-stigma for help-seeking (see Figure 1). We conducted path analysis using the *cfa* function from the *lavaan* package in R (42) and the diagonally weighted least squares (DWLS) estimator of the parameters with bootstrap standard error estimation on 5,000 samples and the alpha error rate of 0.05. According to Hu and Bentler (43), the fit of the model tested to capture the complex relationships between variables (Figure 1) was marginally acceptable, χ2(4) = 6.12, *p* = 0.19, CFI = 0.96, RMSEA = 0.08, 90% CI for RMSEA = 0.00–0.20, SRMR = 0.07.

Table 3 shows the estimates of the parameters in the tested model. Prior personal experience of mental illness statistically significantly predicted anticipated self-stigma for mental illness (β = 0.26; 95% bootstrap confidence intervals for the estimates excluded the value 0). Similarly, prior personal experience of suicidal behavior predicted anticipation of public stigma for suicidal behavior (β = 0.29). The disturbance (error) terms for self-stigma of mental illness and suicidal behavior self-stigma were correlated (ψ = 0.45). Anticipated self-stigma of mental illness statistically significantly predicted anticipated help-seeking self-stigma (β = 0.40). At the same time, the anticipated public stigma of suicidal behavior showed no independent contribution to the anticipated help-seeking self-stigma (β = 0.07).

**Table 3:** Regression coefficients in the tested model.

**Outcome**	**Regressor**	**Estimate**	**Standard error**	**95% BCI**	**Standardized estimate**
SSOMI	Mental health issues	4.24	1.95	0.23, 7.84	0.26
PSSQ	Suicidal behavior experience	7.32	3.00	1.12, 12.85	0.29
SSOSH	SSOMI	0.34	0.12	0.10, 0.55	0.40
	PSSQ	0.04	0.09	−0.13, 0.20	0.07
	Mental health issues~~Suicidal behavior experience^a^	0.12	0.02	0.06, 0.16	0.48
	SSOMI~~PSSQ^b^	40.99	12.79	18.52, 69.40	0.45

*PSSQ, the Personal Suicide Stigma Questionnaire (anticipation form) scale score; SSOMI, the Self-Stigma of Mental Illness scale score; SSOSH, the Self-Stigma of Seeking Help scale score*.

*95% BCI = 95% bootstrap confidence interval for the estimate*.

*^a^Covariance of the two regressors. ^b^Covariance between the disturbances for the two scale scores*.

## Discussion

To our knowledge, this was the first study to examine the different concepts of anticipated self and public stigma and personal experience of mental illness and suicidality as their predictors in suicide prevention professionals.

The prevalence of mental health problems history found in our sample (51%) was slightly lower than in studies of psychologists (27), therapists (23), counseling psychologists (24), and clinical psychologists (33), and the prevalence of suicidality history (37%) was slightly higher than in some other studies (23). Both figures support the position of Good et al.'s (26) that mental health professionals may also be at risk despite their extensive knowledge of suicide and mental health problems (38). The average scores for SSOMI (30.4) and SSOSH (20.2) in our sample were slightly lower than in some other studies [for example, see studies (11, 44)]. Most of these studies included samples (either undergraduate students or community samples) with previous mental health issues. In contrast, our sample included both individuals with and without such experience in our study. In this sample, individuals with prior experience with mental illness had higher SSOMI and SSOSH scores than other participants. Such individuals most likely responded from the perspective of their lived experience, whereas other participants without such experience responded from an anticipatory perspective.

The mean score of the PSSQ in our study was comparable to the mean score obtained by Rimkeviciene et al. (41) in a convenience sample of Australian adults who reported having been suicidal at some point in their lives. In their study, the mean score was 43.9 for those who reported suicidal ideation and 56.2 for those who also reported a suicide attempt. In our sample, the mean PSSQ score (50.13) of participants with suicidal experience (either suicidal ideation or suicide attempt) was in the interval between these two scores. However, the results cannot be directly compared with those of previous PSSQ studies as we used a modified version of the PSSQ, which we assumed captured the anticipated public stigma of suicidal behavior rather than personal stigma.

To summarize, anticipated self-stigma for mental illness and seeking help was lower in our sample than in other studies. At the same time, we made no such observations regarding anticipated public stigma for suicidal behavior. It seems that suicide prevention professionals in general are open to the possibility of experiencing mental health problems themselves and even seeking help but are less open to the possibility of experiencing suicidality. In addition, almost half of the participants with suicidal ideation experiences did not actually seek help for that. This finding may reflect their attitudes of being strict toward their own experiences of suicidality, which may emerge from the high expectations they have of themselves concerning these matters. The finding deserves to be studied further.

Previous studies reported that individuals with mental illness internalize public stigma and develop self-stigma that includes feelings of shame and incompetence (10, 13). People who have experienced mental illness often report feeling devalued and rejected (45). Similarly, our study found that prior personal experience with mental illness significantly predicted the anticipation of mental-health self-stigma (which may be based on the actual experience of such self-stigma). We also found that such stigma predicted help-seeking self-stigma. Professionals' decisions to disclose their problems in the workplace and seek help may be hindered by shame or fear of being judged negatively or of negative effects of disclosing mental health problems on their career and self-image (28, 33). Thus, mental-health self-stigma can be critical in a professional setting because it can negatively impact feelings of professional competence. This can lead to a vicious cycle: A professional may want to maintain the image of a competent professional (to the public, their clients, and their colleagues) and avoid seeking mental health treatment. Delaying help-seeking can have detrimental effects on mental health, including suicidality development. Beside mental-health (anticipated) self-stigma, other barriers to help-seeking for mental health professionals may be important, such as difficulty of finding an acceptable therapist, lack of time or financial resources, or privacy concerns (22).

The notion that professionals may be motivated to maintain an image of being free of mental illness in the eyes of their colleagues is supported by the finding that many stigmatizing attitudes come from mental health professionals themselves (32). The more someone believes and expects that others stigmatize seeking help, the more likely they are to endorse this stigma themselves (46). Health care specialists are often reluctant to seek professional treatment for mental illness (47). They may fear social stigma and have difficulty finding a local provider they trust, or they may try to treat their mood disorder with self-prescribed medications before seeking help. The fact that 58% of invited participants accessed our survey but only 17% completed it may also be due to stigma and lack of recognition of the importance of this issue among professionals.

When asking a colleague for help, both parties may underestimate the severity of the crisis (34). Personal mental health problems must be addressed and highlighted by all professionals. Providers who assess and help health professionals should use the same assessments and interventions used for nonprofessionals (34). Perhaps an even more critical step in addressing suicide prevention professionals would be to examine their suicidal ideation, as our findings suggest that many of those who had suicidal ideation did not seek help.

Our study has several limitations. The most important one was a small sample and low response rate, limiting the generalizability of the findings and resulting in low statistical power and relatively large standard errors. Due to the small sample size, we could not enter in the model other relevant variables, such as gender, age, or years of activity in the field. We found that age did not correlate with different types of anticipated stigma, which was consistent with the results of meta-analysis by Livingston and Boyd (15). Women in our sample showed slightly higher anticipated public stigma of suicidal behavior and self-stigma of mental illness. Future studies should examine more closely the role of gender in predicting different types of stigma in mental health experts.

In addition, we can only hypothesize about the mental health conditions and stigma among professionals who chose not to participate in the study. Several previous studies (23, 24, 33) have found a higher proportion of mental health professionals with experience of mental health problems than in our study. Because our sample was composed of mental health professionals and other professional profiles working in the field of suicide prevention, it is important to further explore the prevalence of mental health problems among suicidologists as a specific group of mental health experts.

We had planned the survey for March 2020, but as soon as we launched it, the COVID-19 pandemic started, which is likely one of the reasons for the limited response rate, as most professionals faced an additional workload due to the pandemic. This could also possibly lead to a biased sample. For example, mental health professionals experienced a greater workload due to increased number of people in need during the pandemic (48), which limited their availability and willingness to engage in other activities, such as participating in research. It is possible that the slightly lower stigma and prevalence of mental health problems in our sample compared with previous studies was found because more resilient experts responded to our invitation.

The study also did not include a comparison group (e.g., the general public or professionals who do not work in the mental health field). Another limitation is the potential mismatch between the constructs the questionnaires were designed to capture (self-stigma) and the used format of the SSOMI and SSOSH items (in terms of anticipated stigma). Future studies should also examine the construct validity of the adapted PSSQ as a measure of anticipated public stigma of suicidal behavior. This was not possible in our study because of the small sample.

Even though there were no notable correlations between years active in the field of suicidology and different types of anticipated stigma, it would be valuable to explore further the relationship between the level of expertise and stigma, e.g., whether more professional experience and knowledge lead to higher self-expectations and thus more difficulty disclosing one's struggle with mental illness or seeking help, or the opposite is true and there is greater openness to revealing one's mental health issues. The relationship between the prior suicidal history and personal stigma of suicidal behavior also requires further investigation, as does the relationship between gender and mental illness and self-stigma of suicidal behavior. In addition, self-stigma concepts and their predictors should be compared between professionals and the general public to identify characteristics specific to professionals. This could contribute to the development of designated anti-stigma programs. Addressing factors that contribute to the development and perpetuation of stigma among suicide prevention professionals and factors that prevent “coming out” and seeking help could reduce the perceived burden when confronting personal mental health problems and, in some cases, possibly be lifesaving.

To conclude, our pilot study indicates that suicide prevention professionals are, like any other population, vulnerable to mental health issues and suicidal behavior. Those who have experience with mental health problems may anticipate higher self-stigma for mental health, and in turn, may have more concerns about disclosure and help-seeking. Thus, our findings suggest that it is crucial to promote the mental health of suicide prevention professionals and raise their awareness of self-stigma, for example, through regular supervision or addressing these issues in the specialization curricula.

## Data Availability Statement

The raw data supporting the conclusions of this article will be made available by the authors, without undue reservation.

## Ethics Statement

The studies involving human participants were reviewed and approved by Commission for Medical Ethics of the Republic of Slovenia (Ref. No. 0120-609/2019/5). The patients/participants provided their written informed consent to participate in this study.

## Author Contributions

SR had the initial idea for the study and contributed to data collection and manuscript writing. DK contributed to online survey development and data pre-processing. KA, KK, and MV contributed to the design of the study and manuscript writing. AP overviewed the research, ran the statistical analysis, and contributed to manuscript writing. All authors contributed to the article and approved the submitted version.

## Funding

The authors acknowledge the financial support from the Slovenian Research Agency (research core funding No. P3-0339 and P5-0110).

## Conflict of Interest

The authors declare that the research was conducted in the absence of any commercial or financial relationships that could be construed as a potential conflict of interest.

## Publisher's Note

All claims expressed in this article are solely those of the authors and do not necessarily represent those of their affiliated organizations, or those of the publisher, the editors and the reviewers. Any product that may be evaluated in this article, or claim that may be made by its manufacturer, is not guaranteed or endorsed by the publisher.
